# Resolution of Giant Coronary Aneurisms in a Child With Refractory Kawasaki Disease Treated With Anakinra

**DOI:** 10.3389/fped.2020.00195

**Published:** 2020-05-07

**Authors:** Alessandro Gambacorta, Danilo Buonsenso, Gabriella De Rosa, Ilaria Lazzareschi, Antonio Gatto, Federica Brancato, Davide Pata, Piero Valentini

**Affiliations:** ^1^Department of Woman and Child Health and Public Health, Fondazione Policlinico Universitario A. Gemelli IRCCS, Rome, Italy; ^2^Istituto di Microbiologia, Universitá Cattolica del Sacro Cuore, Rome, Italy

**Keywords:** kawasaki disease, anakinra, IL-1 receptor antagonist, Coronary aneurysms, personalized medicine, precision medicine, paediatrics 2

## Abstract

Kawasaki disease (KD) is an acute, febrile illness of unknown etiology that mainly affects children under 5 years of age. intravenous immunoglobulin (IVIG), the standard treatment, has reduced coronary involvement to <5%. Patients who do not improve after an initial IVIG have a higher risk of developing coronary arteries aneurysms, and its optimal treatment remains controversial. We present a case of IVIG, steroids, and infliximab-resistant KD in a 9-month-old child, which developed giant aneurysms and was successfully treated with anakinra, a recombinant antagonist of the IL-1 receptor. In our case, the introduction of IL-1 receptor antagonist therapy seems to have blocked the disease from both a clinical and a laboratory point of view. We also noted a very rapid regression of coronary aneurysms passed from giant aneurysms to small ones, or, as in the case of the anterior descending artery, the complete disappearance of the aneurysm formation. We think that our case adds more evidences to the potential role of IL-1RA as therapy in some selected cases of refractory KD, in particular with severe involvement of coronary arteries, although new efficacy trials are needed to better understand the role of Anakinra in these patients.

## Introduction

Kawasaki disease (KD) is an acute, febrile illness of unknown etiology that mainly affects children under 5 years of age. KD is a clinical diagnosis that relies on the recognition of the main clinical findings, since there are no diagnostic tests.

Traditionally, the prevalence of coronary artery aneurysms (CAAs) was 23%, reduced to ≈4% since the introduction of early high-dose intravenous immunoglobulin (IVIG). Therefore, in some cases, the disease course is not interrupted by the administration of IVIG and, in these circumstances, children can develop CAAs. They can lead to myocardial infarction and sudden death (1). In these cases, with the adjunction of steroids, the second IVIG dose is effective in reducing the incidence of CAA (2, 3).

Other treatments are available for patients who do not respond to the second treatment line such as infliximab (4), cyclosporine (5, 6), and plasma exchange (7). In the last years, the use of IL-1 receptor antagonist (IL-1RA) for refractory KD is increasing, but there are no conclusive data on its

usefulness. Only phase I/IIa trials testing safety, pharmacokinetics, and pharmacodynamics are underway (8, 9).

We describe a case of IVIG, steroids, and infliximab-resistant KD of 9 months of life, which developed giant aneurysms and was successfully treated with anakinra.

## Case Presentation

We describe a previously healthy 9-month-old child admitted to the emergency department for 5 days fever, mild conjunctival hyperaemia, and cervical lymphadenopathy.

He was admitted to the pediatric ward where appropriate blood tests (CRP 20 mg/L, mild neutrophil leucocytosis) and a normal echocardiogram were performed. Then, he started intravenous antibiotic therapy. Oseltamivir therapy was started given the positive result of the nasal swab for influenza virus. In the following days, fever disappeared and analytical features normalized.

On day 9, due to a new febrile peak, marked irritability, and CRP increase, a lumbar puncture was performed and the consequent microbiological tests gave a normal result.

**Figure 1:**
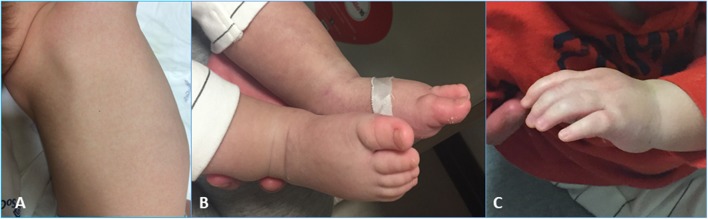
The patient's skin rash **(A)** and edema **(B)** of feet **(B)** and hands **(C)**.

On the same day, due to the appearance of hands and feet edema, cutaneous rash, and scarlet lips (Figure 1), he fulfilled the KD classical criteria; thus, IVIG (2 g/kg) and antiplatelet therapy with Clopidogrel (given the mentioned influenza infection) were started, obtaining a temporary resolution of such symptoms.

On day 14, he presented a new clinical deterioration with fever and high level of CRP. The second dose of IVIG was administered and a new echocardiogram was performed, showing left ventricular ejection fraction of 55% without evidence of CAA.

Due to the development of superficial thrombophlebitis at the left lower limb, anticoagulant therapy with low-molecular-weight heparin (LMWH) was started. It is possible that either KD or a KD-related systemic inflammatory response with consequent development of pro-thrombotic background, along with the presence of peripheral IV line, contributed to the development of superficial thrombophlebitis.

On day 20, since the patient developed a new febrile peak and the inflammation markers increased, methylprednisolone pulses (30 mg/kg for 3 days) were administered, obtaining again a resolution of the symptoms.

**Figure 2:**
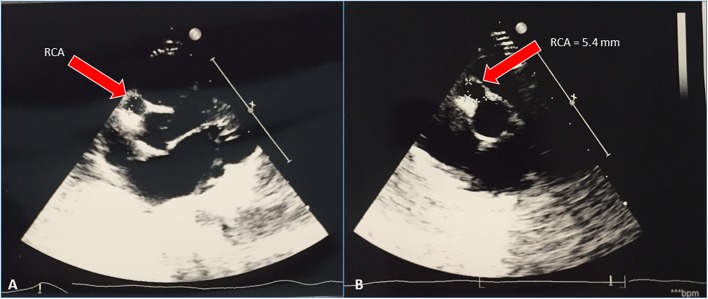
Heart ultrasound showing right coronary artery aneurism (RCA).

Serial cardiac ultrasounds were performed. On day 25, for the first time, the patient developed dilatation of the coronary arteries (Figure 2) with the appearance of small aneurysms involving the left main coronary artery (LMCA), the right coronary artery (RCA), and the left anterior descending artery (LAD).

The next day, the infant presented with a new clinical deterioration, so we decided to infuse infliximab (INFLIXIMAB) (5 mg/kg), obtaining a rapid resolution of fever and decrease of inflammatory markers for 2 weeks.

**Figure 3:**
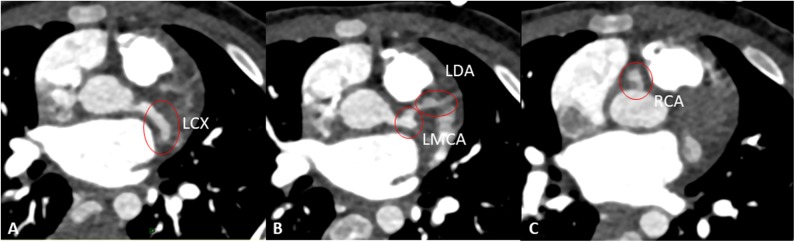
Angio-CT showing aneurisms of the left circumflex artery (LCX, **A**), left main coronary artery (LMCA), and left anterior descending artery (LAD) **(B)**, and right coronary artery (RCA) **(C)**.

Despite the improved clinical conditions, the patient underwent serial echocardiograms that showed a progressive increase of the size of coronary arteries and eventually the development on day 37 of medium aneurysm of LMCA (*Z* score 7.53) and giant aneurysms of RCA (*Z* score 11.49) and LAD (*Z* score 11.2). C.T. Angio confirmed the presence of giant aneurysms and the absence of thrombi (Figure 3).

The patient continued antiplatelet and anticoagulant therapy and started beta-blocker therapy for tachycardia.

On day 40, the infant presented with a new increase of inflammatory markers, marked edema of the hands and feet, and low-grade fever. Therefore, we decided to start further treatment. Current guidelines do not define a specific guide for the treatment of refractory KD and, although Nagatomo et al. suggest that a third infusion of IVIG is possible (10), we decided to try different options since our patient had very low response to the first two doses of IVIG. Thus, treatment by anakinra, IL-1 RA, was started. Parents were informed that there were no extensive data available on the use of anakinra in these situations. They agreed to try this option (and provided written informed consent for the publication of results from this treatment protocol).

Anakinra 6 mg/kg/day subcutaneously once a day was administered. Treatment by anakinra was well tolerated with no adverse effects or complications.

**Figure 4:**
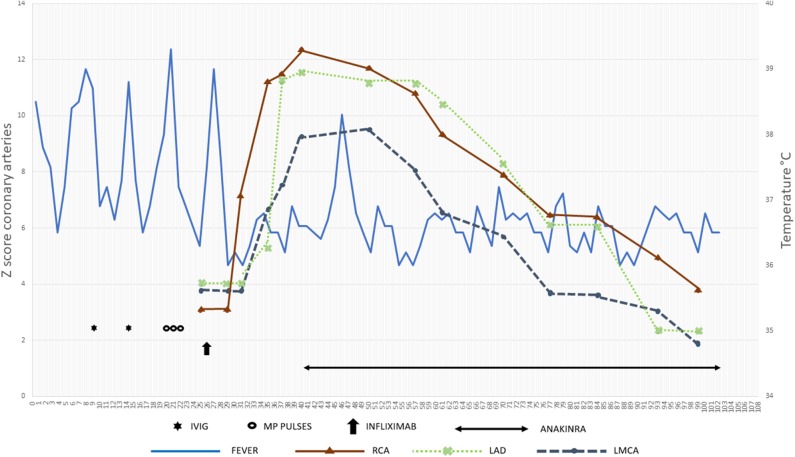
Summary of main data regarding the whole clinical history.

**Figure 5:**
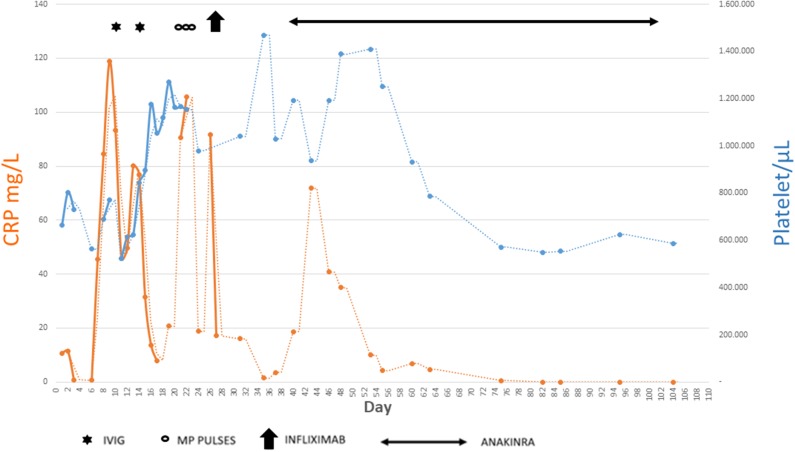
Summary of main inflammatory markers tested during the disease course and how they have been influenced by the different therapies.

In the following weeks, the child presented a progressive improvement of clinical conditions with gradual disappearance of the fever, reduction up to complete normalization of the inflammatory markers and platelet count, and gradual reduction of the size of the coronary arteries: on day 99, RCA was 2.7 mm (*Z* score 3.77), LMCA aneurysm disappeared, and LAD appeared with an uniform ectasia but without aneurysms (Figures 4, 5).

Beta-blocker therapy was administered for 2 months and stopped upon normalization of blood pressure and heart rate; anticoagulant therapy with LMWH was continued for 3 months and stopped due to the absence of giant aneurysm.

Anakinra therapy was continued arbitrarily for 9 weeks and interrupted to avoid side effects, considering the resolution of the clinical symptoms, the normalization of laboratory markers, and the clear improvement of the coronary dilatations.

**Figure 6:**
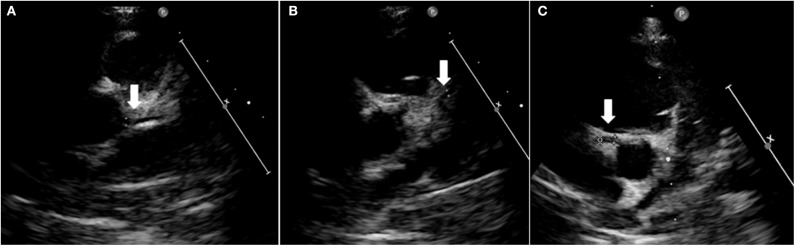
Heart ultrasound showing normal coronary arteries at 1-year follow-up. Left main coronary artery **(A)**, left anterior descending artery **(B)**, and right coronary artery aneurism **(C)**.

The patient subsequently performed follow-up at our hospital that showed stability of the clinical and echocardiographic pattern. At one-year follow-up, cardiac ultrasound unexpectedly showed the complete normalization of coronary arteries (Figure 6).

## Discussion

KD is a systemic vasculitis characterized by increased inflammatory cytokines, such as tumor necrosis factor (TNF)-α, IL-6, and IL-1. The prognosis depends on cardiac involvement and development of CAAs. Although severe myocarditis with hemodynamic instability and shock syndrome can develop in the acute phase of KD, they rarely cause death. The development of CAAs and their sequelae are responsible for the mortality associated with this disease (1).

IVIG represents the standard treatment and have diminished the incidence of coronary involvement to <5%. IVIG should be instituted as early as possible. Conversely, children that do not respond to initial IVIG have a higher risk of developing CAAs (11).

The pathogenesis of IVIG resistance is uncertain, also because the mechanism of action of IVIG is unclear. Researchers think that host genetic factors, such as polymorphisms in the Fc gamma receptors or copy number variation in FCGR2C, FCGR3A, and FCGR3B, can play a role in IVIG response and resistance (12, 13).

The adjunction of steroids to the second IVIG dose seems to be an effective second treatment line but the optimal treatment for refractory KD remains controversial (14).

In our report, the diagnosis was slightly late due to confounding factors such as the documented infection with influenza virus and the initial absence of typical signs of KD such as exanthema, changes in hands and feet, and involvement of the oral mucosa (1). It is possible that starting IVIG on day 5 with a diagnosis of atypical KD would have prevented all the development of further KD-related complications; however, we have no elements to prove it. Importantly, achieving a diagnosis of KD is not always easy and late diagnoses are not infrequent in real-world clinical practice. In these cases, second- to third-line treatment options can be needed; therefore, our case can add more data to available literature and support physicians caring for challenging cases in using uncommon drugs.

Our patient presented a form of KD resistant to multiple treatment lines, particularly the two IVIG infusions and corticosteroid boluses.

Thus, we decide to administer infliximab. KD patients who develop CAA have a defect in expansion of both natural Treg and tolerogenic myeloid dendritic cells (15). Infliximab can downregulate activated monocytes and upregulate Treg toward the normal range (16).

Recently, Gyu Hur (17) and Nagatomo (10) in their retrospective study observed that the early administration of infliximab may reduce the incidence of significant CAA in patients with IVIG-resistant KD.

However, in our case, after infliximab therapy, we obtained a resolution of symptoms more prolonged (14 days) but only temporary and with simultaneous deterioration of the coronary dilation seen in the serial echocardiographic checks. Therefore, we needed an additional treatment line to stop the inflammatory damage.

Anakinra is a recombinant antagonist of the IL-1 receptor currently used to treat systemic onset juvenile idiopathic arthritis (18).

IL-1 is a central mediator of innate immunity and inflammation (19). There are already numerous data about the relationships between innate immunity and IL-1 signaling pathway in KD.

IL-1β signaling prolongs neutrophil survival and drives proliferation of smooth muscle cells (SMCs) and myofibroblast formation, all pathologic hallmarks of the arteriopathy seen in KD. Therefore, these events can increase inflammatory cell infiltration into the arterial wall and cause endothelial cell necrosis (19).

Proteomic and transcriptomic studies in human KD patients demonstrate that the IL-1 plays a role in acute KD (8). Indeed, the IL-1 pathway is upregulated compared to paediatric febrile controls (19) and IVIG treatment reduces the IL-1 β secretion by the peripheral blood mononuclear cells in patients without CAA but not in IVIG-treated patients with CAA in which IL-1 β levels remain elevated (20).

Furthermore, it has already been described in mouse models how the innate response, through the activation of NLRP3, caspase-1, and therefore of the interleukin 1 pathway, is involved in the pathogenesis of KD and in the peculiar remodeling that affects the coronary arteries. The mouse model was obtained by the injection of a cell wall extract from *Lactobacillus casei*, which reproduces arteritis, fever, and the systemic inflammation, typical of KD (21–24).

Using the mouse model of KD, Gorelik and colleagues demonstrated the key role of IL-1 signaling pathways in the mouse vasculitis model and the importance of anakinra in reducing myocarditis and improving heart function (25).

Thus, genetic and transcriptomic data from KD patients and data from the experimental mouse model of KD vasculitis have converged on the likely role of IL-1 signaling in the pathogenesis of KD.

Recently, a lipidomic study has shown that many molecules participate in the pathogenesis of coronary arteritis in KD including oxidized phosphatidylcholine. These data implicate the pathogenic role of atherogenic signals in KD. This suggests that the triggering of the innate response begins not only through the PAMPs or damage-associated molecular patterns (DAMPs) (26) but also from the activation of multiple pathways in a variety of cell types (27).

However, anakinra is not free from side effects such as high risk of infection (including tuberculosis reactivation), injection site reaction, antibody development, neutropenia, diarrhea, nausea or vomiting, and diarrhea. However, given the potential immunological benefits and discussed possible side effects with the family, and having obtained verbal and written consent from the parents, clinicians started to try IL-1 blockade in the treatment of seriously ill KD patients.

In our case, anakinra 6 mg/kg/day subcutaneously once a day was administered for 9 weeks and then interrupted without tapering. We found no side effects related to the drug or disease recurrence.

The peculiarity of this case in our opinion lies in the fact that the introduction of IL-1 RA therapy seems to have blocked the disease from both a clinical and a laboratory point of view. CAAs in KD patients are responsible for morbidity and mortality. In fact, they are associated with numerous complications such as coronary artery stenosis, thrombosis, and myocardial infarctions. There are numerous studies that describe the natural course of CAAs. Kato et al. reported outcomes in 598 KD patients followed up for up to 10–21 years. Aneurysms were diagnosed in 25%, with 49% of these having reduced to a normal luminal dimension 6 to 18 months later. None of them, however, originally had giant aneurysms (28). Akagi et al. more recently showed similar results (29).

In our case, we noted that a very rapid regression of coronary aneurysms passed from giant aneurysms to small ones, or, as in the case of the anterior descending artery, the complete disappearance of the aneurysm formation in just a hundred days. Long-term follow-up (now 1 year after disease onset) showed complete normalization of coronary arteries.

The interleukin-1 pathway promotes the proliferation of SMCs and the formation of myofibroblasts, typical of subacute arterial disease of KD. This proliferation of myofibroblasts can be responsible for the formation of coronary artery stenosis and myocardial infarction (24). The use of IL-1RA, acting on this pathway, could promote the faster resolution of aneurysms and could reduce the probability of coronary artery stenosis formation.

This is the first description with such an important result using anakinra in refractory KD with giant aneurysms. Kone-Pault treated 11 patients with refractory KD obtaining a rapid and sustained improvement in clinical and biological inflammation, but not great results on coronary arteries (30). Similarly, Blonz and colleagues report a case of late-onset KD with prominent coronary involvement and incomplete response to IgIV, which was successfully treated with anakinra but not with complete normalization of coronary abnormalities (31). In both studies, the authors used lower dosages than us, and this may explain the partial response compared to our findings.

We think that our case adds more evidences to the potential role of high-dose IL-1RA as therapy in some selected cases of refractory KD, particularly with severe involvement of coronary arteries, although new efficacy trials are needed to clarify the role of Anakinra in these patients.

## Data Availability Statement

The datasets generated for this study are available on request to the corresponding author.

## Ethics Statement

Written informed consent was obtained from the parents of the participant for the publication of this case report.

## Author Contributions

AGam and DB took care of the patients, were responsible for manuscript writing, and reviewed the final version of the manuscript. AGam, DB, and PV decided the personalized treatment, reviewed the literature about anakinra use in KD, and received final approval by the local pharmacy department for its use. GD and FB performed heart ultrasound, collected heart data, and contributed to manuscript writing. IL, DP, and AGat took care of patient treatment and follow-up and contributed to manuscript writing. PV supervised the study team and revised the final version of the manuscript.

## Conflict of Interest

The authors declare that the research was conducted in the absence of any commercial or financial relationships that could be construed as a potential conflict of interest.
